# Promiscuous RNA Binding Ensures Effective Encapsidation of APOBEC3 Proteins by HIV-1

**DOI:** 10.1371/journal.ppat.1004609

**Published:** 2015-01-15

**Authors:** Luis Apolonia, Reiner Schulz, Tomaž Curk, Paula Rocha, Chad M. Swanson, Torsten Schaller, Jernej Ule, Michael H. Malim

**Affiliations:** 1 Department of Infectious Diseases, King’s College London, London, United Kingdom; 2 Department of Medical and Molecular Genetics, King’s College London, London, United Kingdom; 3 Faculty of Computer and Information Science, University of Ljubljana, Ljubljana, Slovenia; 4 Department of Statistical Science, University College London, London, United Kingdom; 5 Medical Research Council Laboratory of Molecular Biology, Cambridge, United Kingdom; 6 Department of Molecular Neuroscience, UCL Institute of Neurology, Queen Square, London, United Kingdom; University of North Carolina at Chapel Hill, UNITED STATES

## Abstract

The apolipoprotein B mRNA-editing enzyme catalytic polypeptide-like 3 (APOBEC3) proteins are cell-encoded cytidine deaminases, some of which, such as APOBEC3G (A3G) and APOBEC3F (A3F), act as potent human immunodeficiency virus type-1 (HIV-1) restriction factors. These proteins require packaging into HIV-1 particles to exert their antiviral activities, but the molecular mechanism by which this occurs is incompletely understood. The nucleocapsid (NC) region of HIV-1 Gag is required for efficient incorporation of A3G and A3F, and the interaction between A3G and NC has previously been shown to be RNA-dependent. Here, we address this issue in detail by first determining which RNAs are able to bind to A3G and A3F in HV-1 infected cells, as well as in cell-free virions, using the unbiased individual-nucleotide resolution UV cross-linking and immunoprecipitation (iCLIP) method. We show that A3G and A3F bind many different types of RNA, including HIV-1 RNA, cellular mRNAs and small non-coding RNAs such as the Y or 7SL RNAs. Interestingly, A3G/F incorporation is unaffected when the levels of packaged HIV-1 genomic RNA (gRNA) and 7SL RNA are reduced, implying that these RNAs are not essential for efficient A3G/F packaging. Confirming earlier work, HIV-1 particles formed with Gag lacking the NC domain (Gag ΔNC) fail to encapsidate A3G/F. Here, we exploit this system by demonstrating that the addition of an assortment of heterologous RNA-binding proteins and domains to Gag ΔNC efficiently restored A3G/F packaging, indicating that A3G and A3F have the ability to engage multiple RNAs to ensure viral encapsidation. We propose that the rather indiscriminate RNA binding characteristics of A3G and A3F promote functionality by enabling recruitment into a wide range of retroviral particles whose packaged RNA genomes comprise divergent sequences.

## Author Summary

APOBEC3 proteins are cell-encoded restriction factors that counteract infections, particularly by retroviruses such as HIV-1, and retrotransposons. When packaged into HIV-1 particles, APOBEC3G and APOBEC3F both inhibit reverse transcription and induce destructive hypermutation in viral DNA. The mechanism of APOBEC3 virion packaging awaits elucidation, though a dependency on RNA binding has been established. Here, we employed a cross-linking and next generation sequencing approach to determine which RNAs are bound to A3G and A3F in HIV-1 infected cells. We show that both proteins bind to multiple different RNAs, including viral RNA as well as cellular coding and non-coding RNAs, with relatively little evidence of selectivity. We then developed a complementation assay to address the diversity of RNAs that can act as substrates for A3G/F virion packaging. Consistent with the RNA binding profiles, many RNAs can promote packaging provided that those RNAs are, themselves, packaged. These observations suggest that APOBEC3 packaging lacks selectivity and is driven simply by the non-specific RNA binding capabilities of these proteins. We speculate that this model accounts for the broad range of retro-elements that are susceptible to repression by individual APOBEC3 proteins, and also that such substrates cannot escape APOBEC3-mediated inhibition through sequence variation.

## Introduction

The apolipoprotein B mRNA-editing enzyme catalytic polypeptide-like 3 (APOBEC3, or A3) proteins have been identified as potent antiviral effector proteins [1,2]. There are seven family members in humans, each of which contains one (A3A, A3C and A3H) or two (A3B, A3D, A3F and A3G) characteristic zinc-coordination domains, one of which is catalytically active [3]. These proteins have been identified as inhibitors of retroviruses such as human immunodeficiency virus type-1 (HIV-1) [4], simian immunodeficiency viruses, murine leukaemia virus [5–7] and mouse mammary tumour virus [8], as well as viruses from other families such as hepatitis B virus [9], adeno-associated virus [10] and also endogenous retroelements [11]. Viruses have developed assorted strategies to evade A3-mediated inhibition, the most prominent of which is the expression of the dedicated regulatory protein, Vif, by most lentiviruses. Specifically, HIV-1 Vif counteracts APOBEC3 proteins by inducing their proteasomal degradation through the direct recruitment of CBF-β and a cellular E3 ubiquitin ligase comprising CUL5, ELOB/C, and RBX2 [12–15]. When Vif is absent or defective, APOBEC3 proteins are packaged into progeny virions and transferred to target cells during new infections, where they inhibit reverse transcription and hypermutate nascent cDNAs through excessive cytidine-to-uridine editing [5,7,16–19]. Thus, the encapsidation of APOBEC3 proteins into viral particles is essential for their antiviral activity, and a complete description of APOBEC3 protein function will require a full understanding of the packaging mechanism.

APOBEC3 proteins are RNA binding proteins [20–22]. A3G associates in an RNA-dependent mechanism with multiple ribonucleoprotein (RNP) complexes and accumulates in cytoplasmic RNA-rich microdomains such as P-bodies, stress granules and Staufen-containing granules [23–26]. Localisation to these regions does not appear to be important for antiviral function [27,28], and it has been suggested that sequestration in RNPs may be important for downregulation of APOBEC3 protein activity within cells. These findings further raise the question of how APOBEC3 proteins are packaged into assembling virions. One elegant study has demonstrated that it is newly synthesised protein that is encapsidated, presumably by averting entrapment into cytoplasmic RNPs [29].

The packaging of A3G into HIV-1 particles requires the nucleocapsid (NC) region of the viral Gag protein [30–33]. It has been established that the A3G interaction with NC is RNA-dependent, leading to the consensus view that its packaging is reliant upon its capacity to bind RNA [30–33]. However, although several groups have sought to define specific RNAs that are responsible for A3G packaging, a clear consensus has not yet emerged. In particular, Kahn et al. suggested that viral genomic RNA (gRNA) is required for A3G packaging [34], Wang et al. have concluded that 7SL RNA is the responsible RNA [35], and Svarovskaia et al. proposed that both viral and cellular RNAs play a role [32].

A3G interacts with diverse RNAs such as the 7SL RNA (the RNA component of the signal recognition particle, SRP), Alu RNAs, human Y RNAs and several mRNAs [24,36], many of which are also packaged into retroviruses (reviewed in [37]). In our study, we used an unbiased strategy to identify the RNAs that interact with A3G or A3F in HIV-1 infected cells and in budded virions, and then undertook specific experiments to investigate the involvement of such RNAs in A3G and A3F packaging. To define the interacting RNAs, we employed a cross-linking and immunoprecipitation technique (iCLIP) followed by next generation sequencing. This method has successfully defined RNAs that interact with proteins such as neuro-oncological ventral antigen (NOVA), hnRNP C and Argonautes [38–40].

Here, we demonstrate that A3G and A3F interact with a broad range of RNA molecules, including HIV-1 gRNA, cellular mRNAs and a number of small non-coding RNAs. A series of cell-based assays revealed that no single/unique RNA mediates the encapsidation of A3G or A3F, suggesting that multiple, diverse RNAs can recruit APOBEC3 proteins into viral particles, provided that they are themselves packaged. We therefore propose that A3G and A3F exploit their relatively non-specific RNA binding capabilities to patrol the cytoplasm for nascent retroviral RNA, thereby ensuring effective capture by assembling viruses and resultant antiviral function.

## Results

### iCLIP reveals that A3G and A3F bind to diverse RNA targets

Packaging of A3G into HIV-1 particles requires the nucleocapsid (NC) region of p55^Gag^, and it has been established that this interaction is RNA-dependent [30–33]. This led to the consensus view that A3G packaging depends on RNA binding. In order to identify the specific RNAs to which A3G binds for efficient packaging, we first applied an unbiased, deep sequencing method to catalogue the RNAs that are bound to A3G and A3F in living cells productively infected with HIV-1. We also extended this study to determine A3G and A3F target RNAs in cell-free HIV-1 virions.

To generate libraries of RNAs bound to A3G or A3F, we first generated CEM-SS human T-cell lines that stably expressed GFP (negative control), GFP-A3G, GFP-A3F, T7-GFP (negative control), T7-A3G or T7-A3F (S1 Fig.). By using two different immunoprecipitation tags for A3G and A3F, we could identify if either the GFP or T7-epitope tag biased the resulting library. Importantly, our study is distinguished from all others (to the best of our knowledge) by inclusion of GFP-only controls: this is an important addition as it allows the determination of RNA binding enrichment relative to background. These cultures were challenged with *vif*-deficient HIV-1 such that more than 90% of the cells were infected, as judged by intracellular p24^Gag^ staining (S2 Fig.). 48 h after infection, the supernatants were collected and used to assess A3G and A3F antiviral efficacy. Regardless of the tag, both A3G and A3F were antiviral and inhibited single-cycle virus infectivity by 100- and 30-fold, respectively (S3 Fig.).

**Figure 1:**
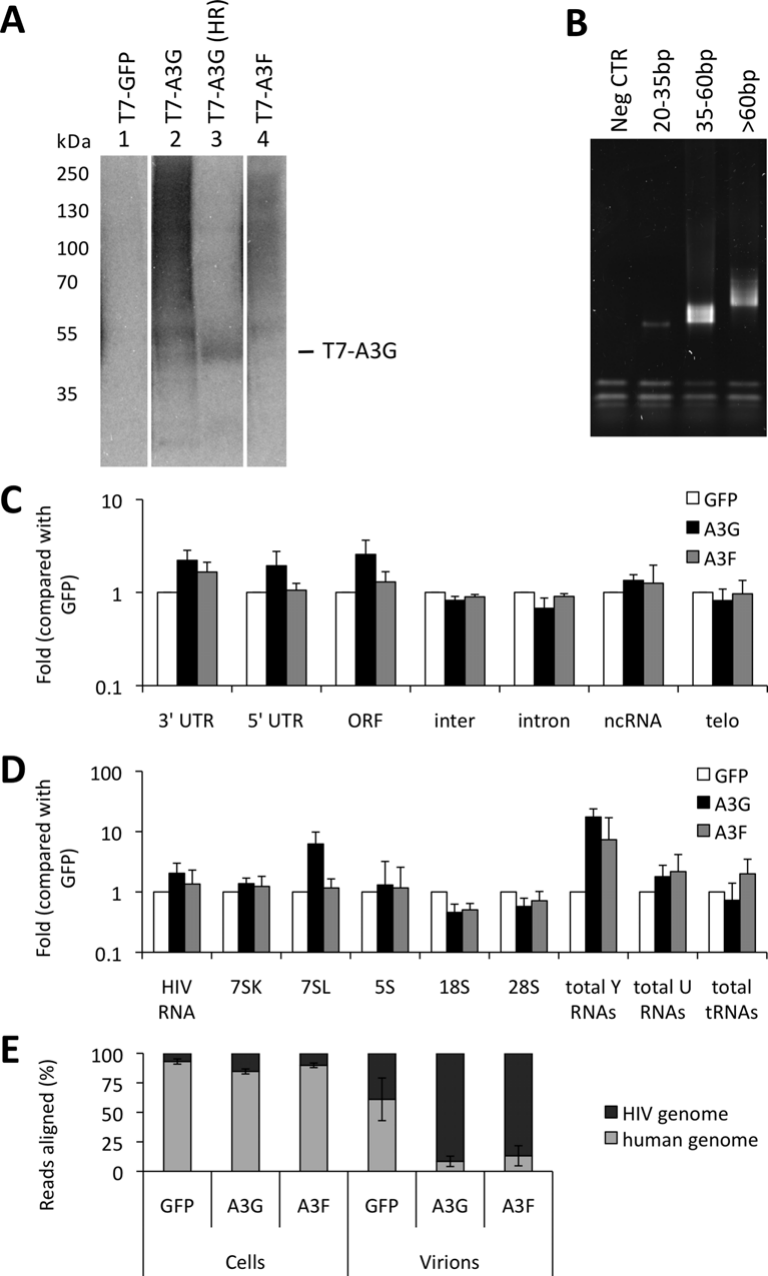
iCLIP reveals which RNAs are bound to A3G and A3F in living cells. (A) CEM-SS T cells stably expressing GFP, GFP-tagged A3G or A3F, or T7-tagged GFP, A3G or A3F were infected with *vif*-deficient HIV-1_IIIB_. Cells were collected 48 h later, subjected to cross-linking with UV and lysed. A high concentration of RNase A was added to one sample (HR, lane 3) and a low concentration to the rest of the samples. Lysates were sonicated and the proteins of interest were immunoprecipitated with anti-GFP or anti-T7 antibodies bound to dynabeads. A linker was ligated to the nucleic acids, and these were radiolabeled with P^32^-ϒ-ATP. The samples were resolved by SDS-PAGE, and the RNA was visualised by autoradiography. A representative gel is shown. **(B)** RNAs running at a higher molecular mass than the proteins of interest were extracted from the membrane and reverse transcribed using a bar coded primer annealing to the previously ligated linker. The cDNAs were multiplexed and run on a TBE-urea gel. Bands ranging from 70–85, 85–110 and >110 base pairs (that contain 20–35, 35–60 and >60 bp of insert) were excised and the nucleic acids were isolated. The cDNAs were circularised, digested with BamHI and amplified by PCR. The product was then run on a gel to assess the quality of the library. A representative library is shown. The fractions that did not contain primer dimers of each library were mixed and sequenced. **(C)** Reads obtained from sequencing were aligned to the human genome. Only reads that aligned once with the possibility of 1 mismatch were considered for further analysis. Reads aligning to each gene were divided by the total number of reads in the library and the relation for each of the replicates was determined (r>0.9) and for each of the differently tagged proteins (r>0.9). The reads were then sorted into categories: 3’-UTR, 5’-UTR, open reading frame (ORF), intergenic regions (inter), intron, non-coding RNAs (ncRNA) and telomers (telo) and the values compared with the GFP negative control. The graph shows the average fold of 4 independent replicates obtained for A3G and A3F compared with GFP for each category of sequence and their respective standard deviations. **(D)** Reads were aligned to the HIV-1 genome as described for panel C. Repeat masker was used to align reads to specific genes that are found in viral particles. Reads aligning to Y1, Y3, Y4 and Y5 RNAs were added and considered as total Y RNAs. Similarly, we considered the same for U RNAs and tRNAs. The average fold compared to GFP of the 4 independent libraries was then plotted with standard deviations. **(E)** Reads obtained in the libraries of HIV-1-packaged A3G and A3F were aligned as described in panel C. The percentage of aligned reads to the human and HIV-1 genomes for the iCLIP performed with cells or virions was then calculated for each sample. Here, we show the average of the 4 independent replicates with standard deviations.

Virus-producing cells and viruses produced from these cells were used to generate iCLIP libraries. Fig. 1A shows RNA cross-linked to the proteins of interest after T7 or GFP directed immunoprecipitation, RNAse digestion (used to shorten the length of bound RNAs for later deep sequencing) and ligation of a linker. Of note, A3G and A3F each cross-link vastly more RNA than GFP, consistent with the fact that these proteins are established RNA binding proteins. The RNAs that migrate at higher molecular weights than the protein of interest were extracted. A primer that anneals to the linker was then used to generate cDNA. The cDNAs were circularised, a further primer annealed over the linker to create a region of double stranded DNA and digested with BamHI. This procedure generated DNAs where the unknown sequence was flanked by linkers, allowing PCR amplification of the library (Fig. 1B) and next generation sequencing.

The sequencing provided a total of 20 million reads of 50 nucleotides each. The libraries obtained for each protein contained between 0.1 and 5 million sequences. We aligned the reads to the human genome and to the HIV-1 genome using bowtie [41]. Only reads that aligned uniquely in the genomes with a maximum of two mismatched nucleotides were considered for analysis. Furthermore, the reads containing the same random barcode and truncated at the same nucleotide were considered PCR artefacts, and only one of such reads was considered. These unique reads represented between 80% and 95% of the total reads for each replicate experiment, demonstrating the high level of sequencing library complexity obtained in the experiments. Human mRNAs were split into regions, specifically the 5’- and 3’-untranslated regions (UTRs), introns and open reading frames (ORFs), and each region type was analysed independently. The number of reads aligning to a specific sequence or class of sequences was divided by the total number of aligned reads obtained from the library and then compared with the GFP control to provide measurements of enrichment. We performed two independent experiments for each cell line. By comparing these replicates, we observed that the data sets were highly correlated (r>0.98). The data obtained from the differently tagged (GFP versus T7) proteins also exhibited high correlation (r>0.95). Therefore, we present here the averaged data obtained for the four libraries with standard deviations. As summarised in Fig. 1C, A3G and A3F binding to mRNAs was enriched compared to the GFP control. While the binding of both A3G and A3F to the 3’-UTRs was higher (2-fold), only A3G was enriched in the ORF or 5’-UTR regions (2- and 3-fold, respectively); Fig. 1C and S1 Table).

We next looked in detail at virion-associated RNA: both the gRNA and host RNAs such as tRNAs, U RNAs, 7SL, ribosomal RNAs and Y RNAs [37]. Because these cellular RNAs are transcribed from repeat elements in the human genome, the reads were aligned using the repeat masker software [42] to a library containing consensus sequences of the elements, allowing misalignment of up to 3 nucleotides per read. As before, hits in each repeat consensus were divided by the total number of reads and compared to the GFP negative control. Fig. 1D and S2 Table show that A3G and A3F bound 2-fold more to the viral gRNA compared with GFP. Also, both A3G and A3F bound to Y RNAs (17- and 7-fold, respectively) and to U RNAs (2-fold), as compared to GFP. Interestingly, A3G bound to 7SL (14-fold) while A3F did not.

As depicted in Fig. 1E, we observed that in infected cells, 20% of the RNA that is bound to A3G or A3F is of viral origin. However, in viral particles this RNA constitutes the majority of the library (80% of reads aligned to HIV-1 gRNA). This implies that, in *vif*-deficient HIV-1, A3G/F may be packaged mainly through interactions with HIV-1 gRNA, or that APOBEC3 proteins bind to encapsidated cellular RNAs and then transfer to gRNA once inside the viral particles.

In light of the diversity of RNA substrates bound by A3G or A3F, we undertook a series of studies designed to determine which RNAs can mediate the encapsidation of A3G or A3F into budding HIV-1 particles.

### HIV-1 genomic RNA is dispensable for A3G and A3F packaging

**Figure 2:**
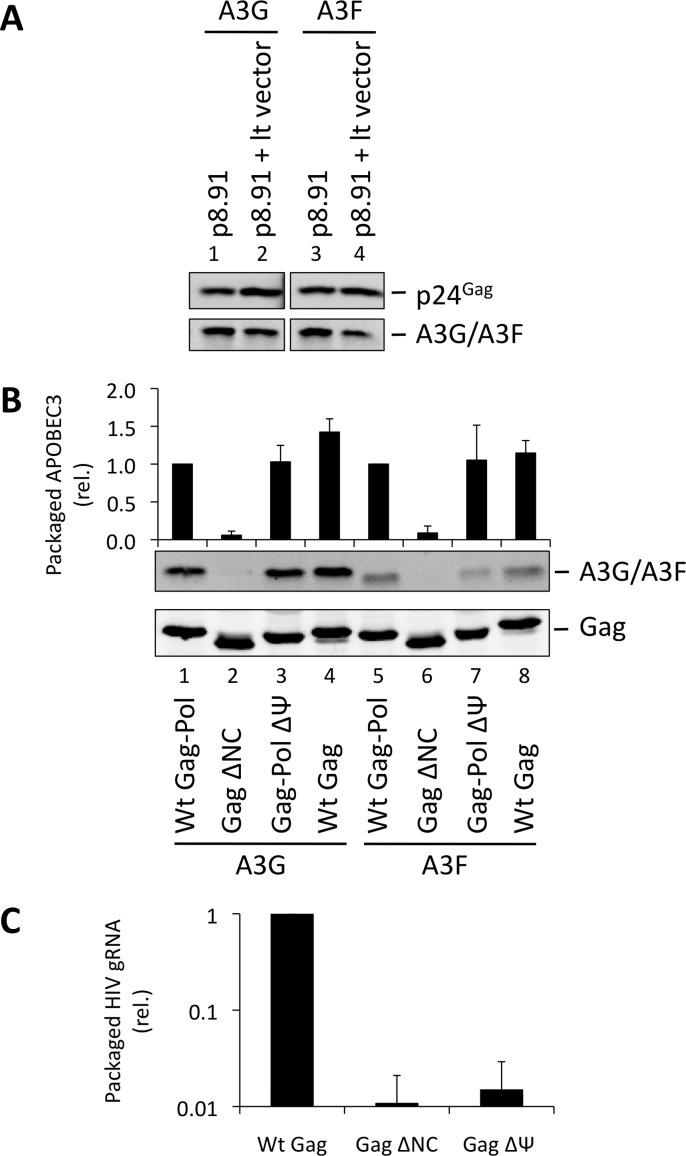
HIV-1 particles lacking genomic RNA package A3G and A3F. (A) 293T cells stably expressing HA-tagged A3G or A3F were co-transfected with the packaging plasmid p8.91, and either with a vector coding for a package-competent lentiviral RNA (lt vector) or a mock plasmid. Vectors were harvested 48 h later and concentrated through a sucrose cushion. Gag proteins and A3G or A3F were visualised by immunoblot using anti-p24^Gag^ and anti-HA antibodies. A representative immunoblot of 3 independent replicates is shown. **(B)** VLPs were produced by expression of the Gag protein of interest in 293T cells expressing HA-tagged A3G or A3F. Particles were concentrated and proteins detected as in panel A. Protein bands were quantified by densiometry using the Li-cor Odyssey infrared imaging and quantification software. Values obtained for A3G/A3F were divided by their respective p24^Gag^ values. A representative immunoblot is displayed, together with a graph showing the average and standard deviation obtained for at least 3 experiments. **(C)** RNAs were harvested from VLPs, and genomic RNA was quantified by qPCR. The value obtained for Wt Gag-Pol VLPs was set to 1 and the other values compared to that. Values and error bars represent the average of 3 experiments and the standard deviation, respectively.

One obvious RNA species that could potentially mediate the packaging of A3G into particles is the viral gRNA. Indeed, at least one previous report has considered this RNA to be essential for A3G packaging [34]. Since A3G and A3F are clearly able to bind to this RNA in infected cells, we performed packaging assays to test this hypothesis. First, we used lentiviral vectors with or without gRNA (Fig. 2A). Succinctly, 293T cells stably expressing HA-tagged A3G or A3F were co-transfected with the HIV-1-based packaging plasmid (p8.91) [43], the VSV glycoprotein envelope expression vector, and either the pHR’SIN-cPPT-SEW lentiviral vector plasmid (denoted lt vector) [44] that expresses gRNA with an intact packaging signal (Ψ) or a mock plasmid. Immunoblot analysis of particles harvested 48 h post transfection and isolated through a sucrose cushion shows that A3G and A3F were packaged into both viral vectors with almost identical efficiency, indicating that viral gRNA is not required for effective A3G or A3F packaging.

These data were next confirmed using an alternative experimental system where the gRNA also serves as the mRNA for Gag (Fig. 2B). 293T cells stably expressing HA-tagged A3G or A3F were transfected with expression vectors encoding: (i) Gag-Pol, (ii) Gag, (iii) Gag in which NC had been replaced by a leucine zipper domain (Zwt) to allow for Gag multimerisation and VLP production (Gag ΔNC) [45], or (iv) a Gag-Pol vector in which the previously defined gRNA packaging determinant between SL2 and SL3 [46] had been deleted (ΔΨ); (S4 Fig.). The protease region of Pol was rendered inactive in the Gag-Pol constructs to facilitate the detection and comparison of Gag across the different samples. Analysis of viral-like particles (VLPs) confirmed that NC is necessary for efficient A3G and A3F packaging, but showed that the Ψ element is dispensable (Fig. 2B, lanes 3 and 7) [30–33]. Since Gag alone was able to package A3G or A3F efficiently, we conclude that Pol is also not required for the packaging of A3G and A3F (Fig. 2B, compare lanes 4 with 1 and 8 with 5).

We also determined the amount of gRNA packaged into wild type Gag, Gag ΔNC and Gag-Pol ΔΨ VLPs by quantitative real time PCR (qRT-PCR) analysis (Fig. 2C). As anticipated, the Gag ΔNC, and ΔΨ VLPs each contained <5% of the level of gRNA compared to wild type Gag VLPs.

### 7SL is not necessary for A3G or A3F packaging

We next examined a second RNA, the non-coding 7SL RNA of the SRP, for its importance in A3G/F packaging. This RNA is incorporated into retroviral particles [47–49]. In particular, our iCLIP analyses (Fig. 1D) confirmed earlier work showing that A3G, unlike A3F, binds to 7SL RNA [50]. Of note, one group has previously reported that 7SL is the RNA required for A3G packaging [35], whereas a second concluded the opposite [51].

**Figure 3:**
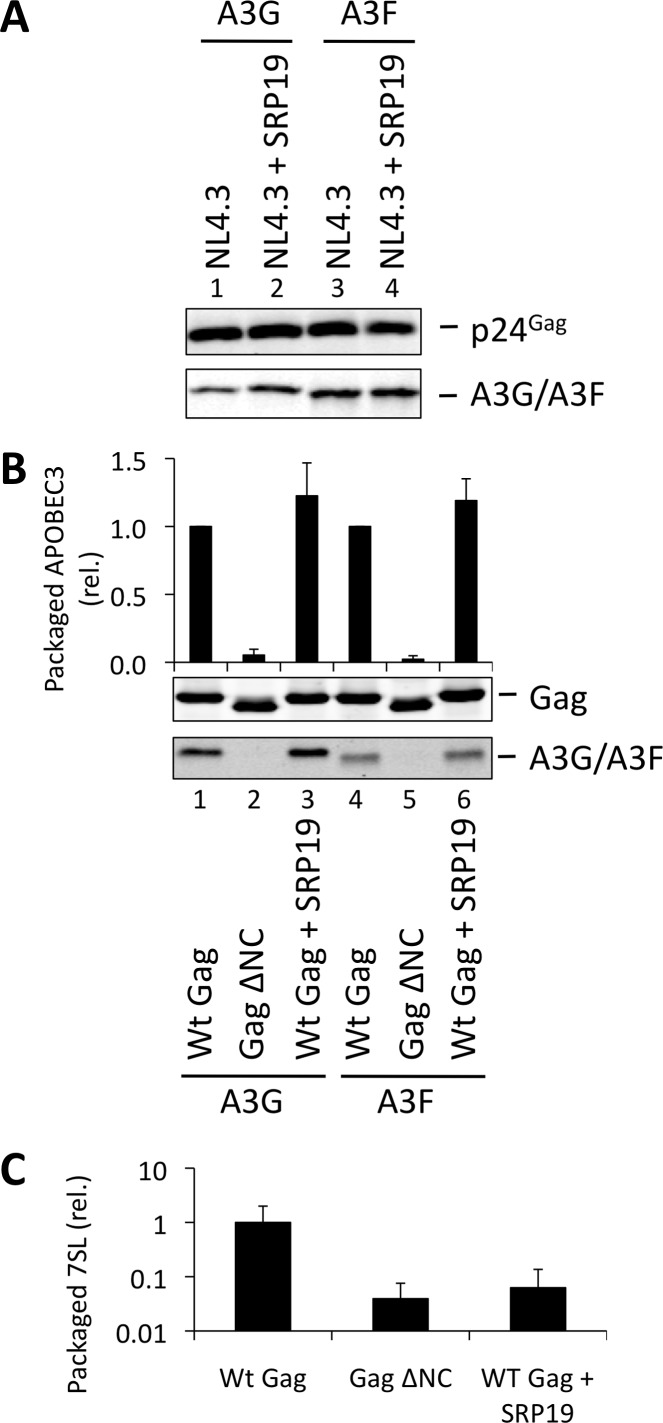
VLPs with reduced 7SL RNA content package A3G and A3F. (A) The *vif*-deficient NL4-3 provirus was co-transfected with a plasmid expressing SRP19 or a control plasmid into 293T cells stably expressing HA-tagged A3G or A3F. Viruses were harvested 48 h later and concentrated through a sucrose cushion. Gag and A3G/F were detected by immunoblot with representative data from one of at least 3 independent experiments shown. **(B)** 293T cells stably expressing HA-tagged A3G or A3F were co-transfected with Gag expression constructs and with a plasmid expressing SRP19 or an empty vector. VLPs were harvested 48 h later and concentrated through a sucrose cushion. Gag proteins and A3G or A3F were visualised by immunoblot. Proteins in VLPs were quantified as in Fig. 2B. The graph shows the average of 3 independent experiments and the respective standard deviation. **(C)** RNAs were extracted from VLPs and 7SL RNA was quantified by qPCR. The average and standard deviation of 3 experiments are shown, where the value obtained for Wt Gag was set to 1 and the others compared to it.

Over-expression of SRP19, a protein component of the SRP, reduces the amount of 7SL RNA packaging into HIV-1 particles [35], presumably by binding to free cellular 7SL RNA and precluding its interaction with Gag and resultant incorporation into assembling virions. Accordingly, we transfected 293T cells stably expressing A3G or A3F with a plasmid encoding the *vif*-deficient NL4-3 provirus together with an expression vector for SRP19 or a control vector. Immunoblot analysis 48 h post transfection showed that both A3G and A3F were still packaged into virions, irrespective of the reduction in virion-associated 7SL RNA (Fig. 3A). Interestingly, reducing the amount of packaged 7SL did not influence the infectivity of the viruses or the antiviral activity of the packaged A3G or A3F (S5 Fig.). The data were then confirmed using Gag VLPs, with Gag ΔNC serving as a negative control (Fig. 3B). Quantitative RT-PCR analysis of RNA extracted from these VLPs confirmed that SRP19 overexpression reduced virion-associated 7SL RNA levels by more than 90% (Fig. 3C). Our data therefore rule out a unique requirement for 7SL RNA for efficient A3G or A3F incorporation into virions, suggesting that their packaging may be mediated by other RNAs.

**Figure 4:**
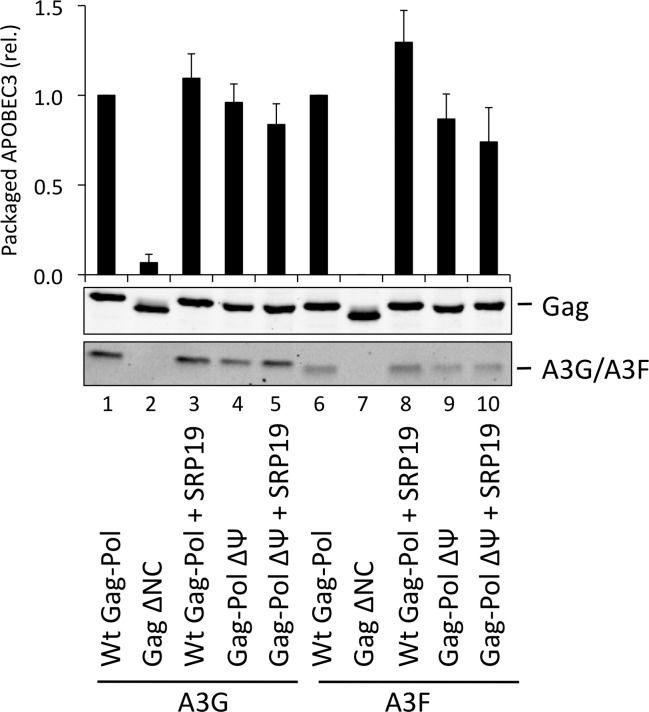
A3G and A3F are packaged into VLPs doubly depleted for genomic RNA and 7SL RNA. VLPs were produced in 293T by co-transfection with expression vectors for HA-tagged A3G or A3F and a plasmid expressing Gag-Pol, Gag-Pol ΔΨ or Gag ΔNC. SRP19 (or a negative control) was over-expressed where indicated. Gag and A3G/F proteins in concentrated VLPs were visualised by immunoblot. Proteins in VLPs were quantified as described in Fig. 2A. The average of 3 replicates with standard deviation is shown here with a representative immunoblot.

To investigate a potential redundancy between HIV-1 gRNA and host 7SL RNA, we next transfected 293T cells expressing A3G or A3F with a vector encoding SRP19 to inhibit 7SL packaging, as well as the aforementioned ΔΨ construct to generate VLPs depleted of gRNA. We observed that A3G and A3F were both packaged with normal efficiencies irrespective of SRP19 over-expression and/or the prevention of gRNA packaging (Fig. 4). Our observations imply that APOBEC3 proteins are packaged into these particles through the action of other RNAs.

### Gag fusion proteins demonstrate that 7SL RNA and Y RNAs can promote APOBEC3 protein packaging

Our data suggested that a variety of host cell RNAs might be involved in APOBEC3 protein packaging into virions. To investigate this further, we next devised an alternative experimental approach whereby the packaging of RNA was dictated by heterologous RNA binding proteins (or domains thereof) rather than by the product of binding to NC and intracellular abundance. To do so, RNA binding domains were genetically fused to the carboxy-terminus of Gag ΔNC, and these proteins were used to generate VLPs in the presence or absence of A3G or A3F. In case of poor virion production, Gag ΔNC was co-expressed to ensure efficient VLP production [52,53].

**Figure 5:**
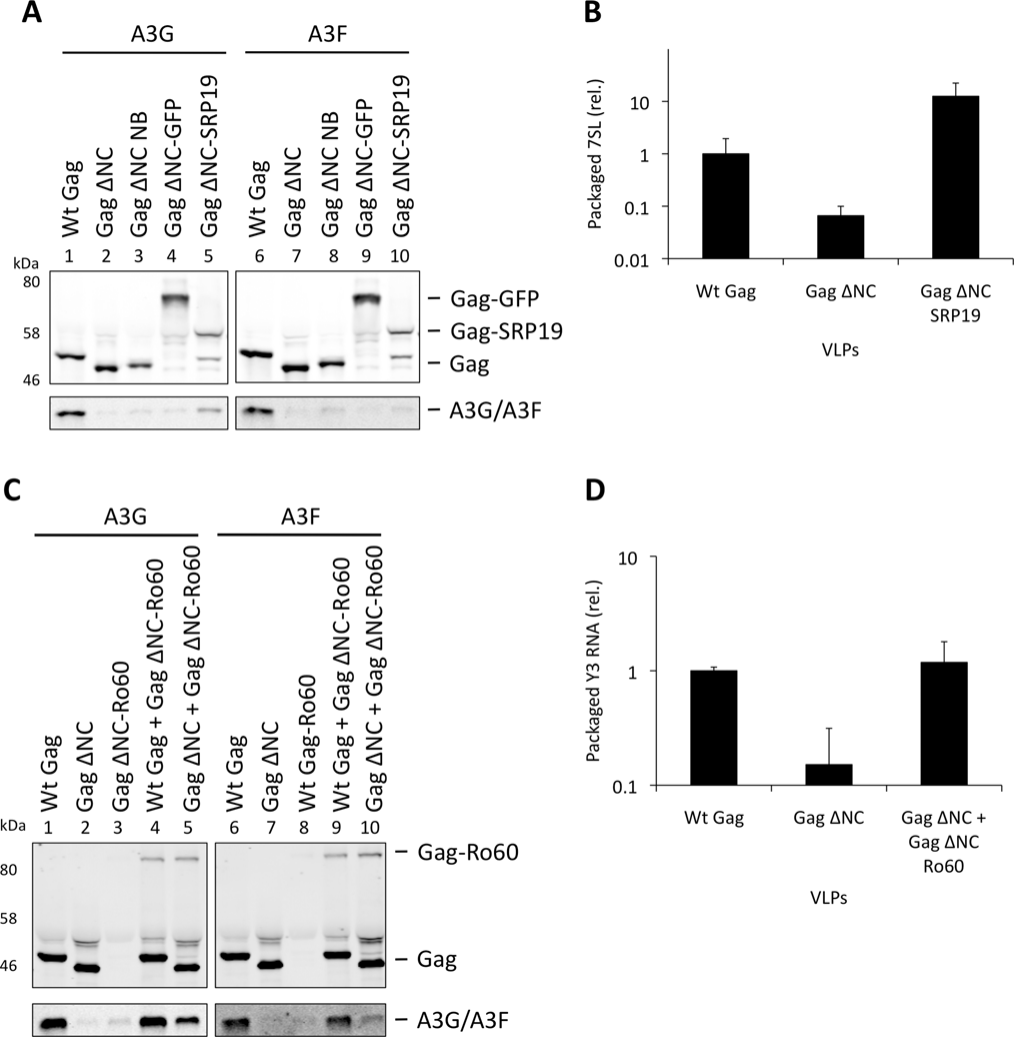
Incorporation of A3G and A3F into Gag-SRP19 and Gag-Ro60 containing VLPs. (A) 293T cells were co-transfected at a 1:5 ratio with HA-tagged A3G or A3F expression vectors and Wt Gag or the different Gag ΔNC constructs indicated. Gag and A3G/F proteins from concentrated VLPs were then visualised by immunobloblot. **(B)** RNA was extracted from VLPs and 7SL RNA was quantified by qPCR. The average of 3 replicates with standard deviation is shown. **(C)** Expression vectors for HA-tagged A3G or A3F were co-transfected with Gag constructs at a ratio of 1:5 into 293T cells. Where indicated, Wt Gag or Gag ΔNC were co-transfected with Gag ΔNC fused to Ro60 at a ratio of 1:5 but maintaining the overall levels of Gag expression plasmid between samples. VLPs were harvested and concentrated. Gag and A3G/F were visualised by immunoblot. A representative result from 3 independent experiments is shown. **(D)** VLPs were lysed and the RNAs extracted. Y3 RNA was detected by qPCR. The average of 3 replicates with standard deviation is shown.

To verify the experimental system, we initially expressed SRP19 fused to Gag ΔNC, anticipating that SRP19 would bind 7SL RNA and recruit it into VLPs together with A3G or A3F. Fig. 5A demonstrates that the addition of 6 amino acids to the C-terminus of Gag ΔNC, creating convenient restriction sites to allow fusions to Gag ΔNC (lanes 3 and 7), does not mediate APOBEC3 packaging. Also, A3G/F packaging was not mediated by the fusion of Gag to GFP, a protein that does not specifically bind to RNA (lanes 4 and 8). Although SRP19 was cleaved from Gag (lanes 5 and 10), the produced particles still contained intact Gag ΔNC-SRP19. Quantification of the amount of 7SL RNA in these VLPs demonstrated that the presence of SRP19 enables the efficient packaging of 7SL RNA (Fig. 5B). Importantly, these Gag ΔNC-SRP19 particles were able to package A3G efficiently, but not A3F. This observation is in agreement with the iCLIP data, which show that A3F does not preferentially bind to 7SL. Thus, while 7SL RNA is not required for the packaging of A3G into HIV-1 particles with an intact NC domain (Fig. 3), it is evidently able to promote packaging of A3G when selectively captured by VLPs.

Our iCLIP data suggested that both A3G as well as A3F can bind to Y RNAs, small non-coding RNAs of ∼100 nucleotides that are components of RoRNPs [54,55] (Fig. 1D). These RNAs are also incorporated into HIV-1 particles [35,49]. To investigate their role in A3G/F packaging, we first tried to “knock down” their levels using siRNA mediated silencing. Although we could reduce cellular Y RNA concentrations, the levels that were encapsidated remained unaltered, in line with previous findings where cellular Y RNAs were reduced following RNAi-mediated Ro60 depletion but packaging into MLV particles was unchanged [56].

We then used our established Gag ΔNC fusion system to ask if Y RNAs would be able to recruit A3G or A3F into viral particles. Ro60 was fused to Gag and 293T cells were co-transfected to express HA tagged A3G or A3F with wild type Gag, Gag ΔNC, Gag ΔNC-Ro60, Gag ΔNC-Ro60 together with wild type Gag or with Gag ΔNC. VLPs were produced and immunoblotting showed that, although Gag fused to Ro60 alone did not produce detectable quantities of particles, mixed particles containing the Gag-Ro60 fusion protein and either Wt Gag or Gag ΔNC were formed (Fig. 5C). Quantitative RT-PCR specific for Y3 RNA was performed on RNA extracted from these particles; both mixed Gag ΔNC + Gag ΔNC-Ro60 and wild type Gag particles each contained ∼7-fold more Y3 RNA compared to Gag ΔNC VLPs (Fig. 5D), thus validating our approach. Analysis of the A3G/F contents demonstrated a strong restoration of packaging for the Gag ΔNC + Gag ΔNC-Ro60 mixed particles, though the levels did not match those noted with wild type Gag VLPs (Fig. 5C). These results indicate that controlled packaging of specific RNA ligands of A3G or A3F can determine their encapsidation, presumably by bridging between assembling Gag fusion proteins and APOBEC3 proteins, further demonstrating that specific RNAs can promote the encapsidation of APOBEC3 proteins.

### Diverse RNA-binding domains are able to promote the packaging of A3G and A3F

Having shown that specific RNAs can recruit APOBEC3 proteins into assembling HIV-1 particles, we next asked whether this was a general property of packaged RNAs. To address this, a series of RNA binding domains (RBDs) of cell-encoded RNA binding proteins known to possess broad RNA binding capabilities were fused to Gag ΔNC as above. Specifically, we used the RBDs from two heterogeneous ribonucleoprotein (hnRNP) proteins: hnRNP C1, that binds to uridine tracts of RNAs [39], and hnRNP K, which is the prototypic protein for the KH RNA binding motif and has high affinity to poly(C) RNA [57,58]. We also fused the splicing factor SRSF2 that has a degenerate RNA binding sequence motif [59] and the double stranded RNA binding protein Staufen-1 [60,61].

**Figure 6:**
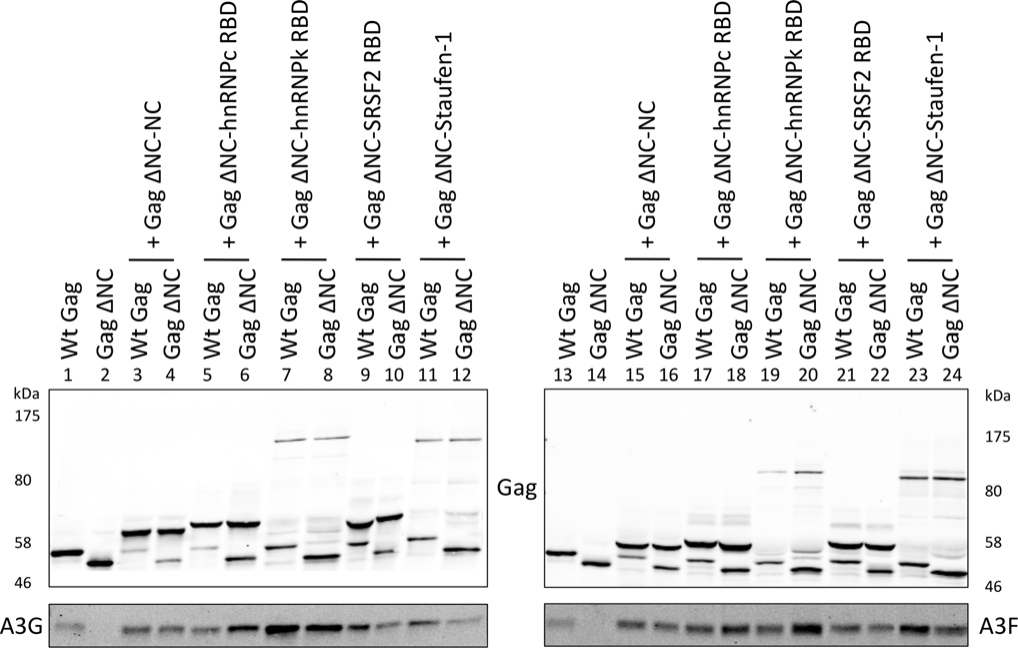
A3G and A3F are incorporated in VLPs when Gag is fused to different RNA-binding domains. Gag ΔNC was fused to RNA-binding domains (RBD) of hnRNP C1, hnRNP K, SRSF2 and Staufen-1. These constructs were co-transfected into 293T cells with vectors expressing either Wt Gag or Gag ΔNC at a ratio of 5:1 and also with HA-tagged A3G or A3F. VLPs were harvested and concentrated, and proteins were visualised by immunoblot. A representative blot of 3 independent experiments is shown.

These expression constructs were co-transfected with A3G/F into 293T cells together with vectors for wild type Gag or Gag ΔNC, and VLPs analysed by immunoblot (Fig. 6). Remarkably, all four RBDs readily rescued packaging of A3G and A3F in mixed virions with Gag ΔNC with (generally) similar efficiency as the wild type Gag or NC itself when reconnected to Gag ΔNC (compare lanes 6, 8, 10 and 12 with 1, 2 and 4; and 18, 20, 22 and 24 with 13, 14 and 16). Given that A3G and A3F exhibit very broad RNA binding characteristics (Fig. 1), we conclude that a multitude of such RNA substrates, if packaged, can serve to draw A3G/F into VLPs.

### A3G and A3F do not preferentially bind to packaged RNAs

**Figure 7:**
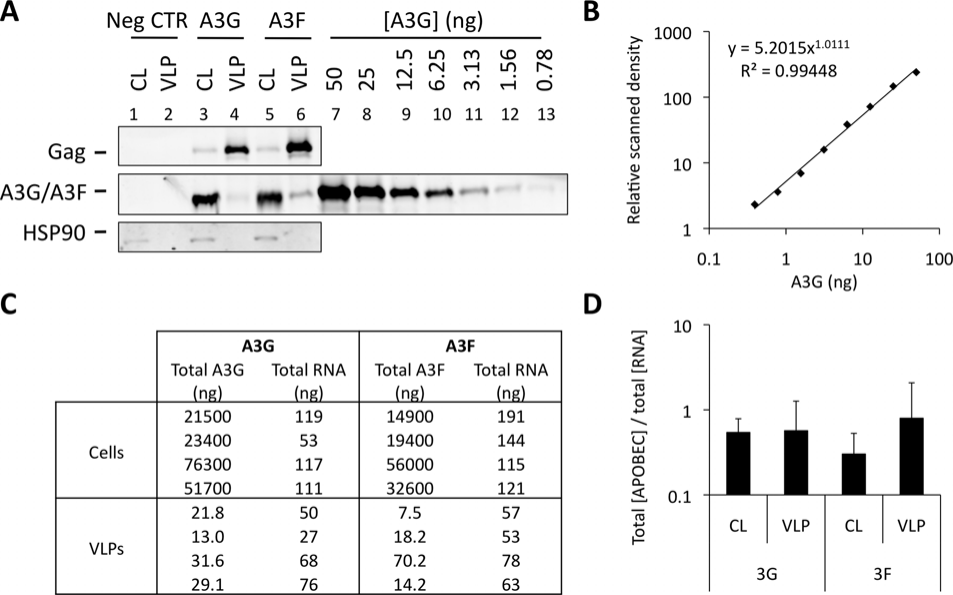
A3G and A3F concentrations are similarly distributed between cells and VLPs by RNA. (A) 293T cells were co-transfected at a 1:5 ratio with expression vectors for T7-tagged A3G or A3F, and Wt Gag. Cell lysates (CL) were kept for analysis. Supernatant from cells transfected with an empty plasmid (negative control) and VLPs were harvested and isolated by ultracentrifugation through a continuous sucrose gradient (20–60%). Fractions were harvested and p24^Gag^-containing fractions were identified by immunoblot. T7-tagged A3G was purified and quantified as specified in the material and methods. Cell lysates, fractions containing VLPs (or the corresponding fraction from the negative control) and purified A3G were analysed by immunoblot. Gag and HSP90 were visualised using anti-p24^Gag^ and anti-HSP90 antibodies, respectively. A3G and A3F were visualised using an anti-T7 antibody. APOBEC3 proteins were quantified by densiometry. This figure shows a representative example. **(B)** Standard curve of purified T7-A3G visualised and quantified by densiometry of the immunoblot of Fig. 7A. **(C)** RNA was extracted from cell lysates (CL) and VLP samples and quantified. Total A3G/F protein in CL and VLPs was quantified using a standard curve. The RNA in the negative control was below the detection threshold of the method. **(D)** The total amount of A3G or A3F was divided by the total concentration of extracted RNA. The graph shows the average of 4 independent experiments. Error bars indicate standard deviation. There was no statistically significant difference between the ratios in lysates and VLPs (p>0.5).

Fig. 6 shows that A3G and A3F can be incorporated into VLPs when diverse RBDs are fused to Gag. This is consistent with our iCLIP data, demonstrating that A3G and A3F are able to bind to multiple diverse RNAs. One obvious question that is raised by these observations is whether A3G/F are preferentially encapsidated by assembling HIV-1 VLPs, or whether they are inevitably packaged as a natural consequence of their promiscuous RNA binding capabilities. To address this directly, we carefully quantified the ratios of A3G and A3F to RNA in the lysates of virus producing cells and the matching particle preparations (Fig. 7). Accordingly, 293T cells were transiently transfected with vectors expressing T7-tagged versions of A3G or A3F, as well as the wild type Gag expression vector. Cells were also transfected with an irrelevant plasmid to serve as a negative control. VLPs and cell lysates were collected at 48 h post transfection. VLPs were isolated through a continuous sucrose gradient and the fractions containing VLPs were identified by immunoblot (S6 Fig.). Protein quantities were then determined against a standard curve of recombinant T7-A3G (Fig. 7A and 7B), and RNA in cell lysates and VLPs were extracted and quantified by Qubit (Fig. 7C). Culture supernatant from cells transfected with an irrelevant plasmid was used to assess background, and RNA was not detected in these samples (threshold of detection, 20 pg/ml). Interestingly, the calculated ratios of A3G/F to RNA were similar in cells and in virus particles (Fig. 7D, mean of 4 independent experiments). In other words, there is no evident enrichment of A3G or A3F in virions relative to virus-producing cells. Taking all our findings together leads us to conclude that the packaging of A3G and A3F into HIV-1 particles is driven by RNA binding, and that multiple/diverse RNAs can fulfil this role provided they are themselves packaged.

## Discussion

A3G and A3F are antiviral proteins that have to be packaged into newly synthesised retrovirus particles to exert their activity. However, the details of the packaging mechanism remain incompletely understood, though a preference for encapsidating newly synthesised A3G has been established [29–35,47,48,51]. Here, we used for the first time a high throughput method to identify the RNAs that these two proteins bind to in living cells productively infected with *vif*-deficient HIV-1 and in cell-free viral particles. We then systematically addressed which RNAs are either necessary or sufficient for A3G and A3F incorporation into budding virions.

An important innovation with our study was the employment of a reference (“non-RNA binding”) protein during the iCLIP procedure, in this case GFP. This enabled us to identify A3G/F RNA ligands that are enriched over background RNA associations that are a presumed property of any protein (Fig. 1; exemplified here by the generation of iCLIP libraries from GFP-containing cells). While some preferential binding to certain classes of RNAs was apparent, it was nonetheless evident that the patterns of A3G/F binding were mostly non-discriminatory. We speculate that such promiscuity in RNA binding could be explained by the capacity of A3G/F to interact with myriad RNA sequences that are available for binding simply because they are not already occupied by other proteins.

We investigated in detail some specific RNAs that are found in retroviral particles [37]. We found that A3G and A3F do not require HIV-1 gRNA or 7SL for packaging into HIV-1 particles (Fig. 2, 3 and 4). However, using our Gag ΔNC fusion assay, we could show that A3G (but not A3F) can utilise 7SL to be incorporated into viral particles (Fig. 5). These observations correlated well with our iCLIP data, where it was shown that A3F does not bind to 7SL.

In accordance with previous studies [30–33], we observed that A3G and A3F are not packaged into Gag ΔNC VLPs. Importantly, these particles can clearly incorporate APOBEC3 proteins when a variety of unrelated RNAs are recruited into assembling virions via fusions of Gag ΔNC to a series of unrelated RBDs (Fig. 5 and 6). In other words, under experimental conditions, A3G and A3F can be packaged into HIV-1 particles via interactions with diverse and unrelated RNAs. Our data also show that 80% of the RNA sequences that A3G/F bind to inside *vif*-deficient (but otherwise normal) viral particles are of viral origin. If we assume that this distribution correlates with the binding of A3G/F to RNAs that are being packaged during particle production, our data imply that viral genomic RNA ordinarily mediates A3G/F packaging. An alternative possibility is that non-viral RNAs recruit A3G/F into particles, but that A3G/F then release these RNAs and bind to gRNA following particle formation and release.

Lastly, fastidious quantification of A3G/F and total RNA levels in cells and budded virions revealed that viral particles are not enriched for APOBEC3 protein content (Fig. 7). While RNA binding is clearly required for APOBEC3 protein packaging, these observations indicate that there is no selectivity for engaging RNAs that are destined for incorporation into assembling viruses. Accordingly, we speculate that cytoplasmic APOBEC3 proteins exploit their relatively non-specific RNA binding capabilities to patrol the cytosol and bind to unoccupied sites on RNAs. In the context of cellular RNAs, this may account for the pronounced accumulation of APOBEC3 proteins in RNA-rich microdomains such as P-bodies and stress granules [23–26]. For viruses with RNA genomes, such as retroviruses, this can result in encapsidation into newly formed viral particles. Given the DNA editing function of APOBEC3 proteins, viruses that have RNA genomes and replicate via DNA intermediates—namely retroviruses and hepadnaviruses—will be susceptible to hypermutation and inhibition [4,5,9]. Indeed, we propose that the relatively non-specific RNA binding characteristics of A3G/F render these proteins well suited to the inhibition of a wide variety of viral and transposon targets. Moreover, this feature may further ensure that viral sequence variation, a noted hallmark of HIV-1, will not afford a means of escape from APOBEC3-mediated restriction: perhaps this underlies the evolution of an entirely different (protein-based) evasion mechanism, namely Vif-induced protein degradation?

## Materials and Methods

### Plasmids and cells

cDNAs encoding SRP19, GFP, A3G or A3F were cloned between the XbaI and BamHI sites of pCGTHCF_FL_T7 [62]. DNA fragments encoding T7-tagged derivatives of GFP, A3G or A3F were cloned between the XhoI and EcoRI sites of the retrovirus vector, pCMS28 [26]. A GFP-containing fragment with a GST linker sequence at its 3’-end was cloned between the EcoRI and XhoI restriction sites of pCMS28, and A3G or A3F cDNAs were then inserted using the NotI and XhoI sites. Plasmids expressing HA-tagged A3G and A3F were previously described [50]. *vif*-deficient HIV-1_NL4-3_ [27] and HIV-1_IIIB_ [63] strains were used where indicated. The wild type Gag-Pol vector, pCMS446, was generated by inserting the HIV-1 5’ UTR-Gag-Pol, Protease activity inactivated (nucleotides 455–5096 from the HV-1_HXB2_ isolate [GenBank: K03455.1] [64]) fragment into pcDNA3.1 (Invitrogen) that contains the HIV-1 RRE [65]. pGag was generated by deleting the Pol sequence 3’ to the Gag stop codon. pGag ΔNC was generated by cloning the analogous 5’ UTR-Gag-Pol fragment from the Zwt-p6 proviral construct [45] into the same RRE-containing vector. SL2 and 3 were deleted from pGag by overlapping PCR using the primers 5′-AGGGGCGGCGACTGGTGAGAGATGGGTGCGAGAGCGTCAGTATTAAGC-3′ and 5′-TGACGCTCTCGCACCCATCTCTCACCAGTCGCCGCCCCTCGCCTCTTGC-3′ to generate pGag ΔΨ [46]. pGag ΔNC NB was created by inserting NheI and BamHI restriction sites in frame 5’ to the stop codon of Gag in pGag ΔNC. NC (HIV-1_HXB2_ strain), GFP, SRP19, Ro60, hnRNP C1 (amino acids 1–104), hnRNP K (amino acids 38 to 464 with amino acids 323–338 deleted), SRSF2 (amino acids 2–93) and Staufen 1 cDNAs were inserted into pGag ΔNC NB using the NheI and BamHI sites.

293T and HeLa cells were obtained from the American Tissue Culture Collection (ATCC). TZM-bl cells were obtained through the NIH AIDS Reagents Repository Program (ARRP). These cell lines were cultured in Dulbecco’s modified Eagle’s medium (Invitrogen, UK) supplemented with 10% foetal bovine serum and 1% penicillin/streptomycin. CEM-SS T cells, from ARRP, were cultured in Roswell Park Memorial Institute 1640 medium (Invitrogen, UK) supplemented with 10% foetal bovine serum and 1% penicillin/streptomycin. Stable CEM-SS T cell lines were generated by standard retroviral transduction using MLV-based vectors expressing GFP, GFP-A3G GFP-A3F, T7-GFP, T7-A3G or T7-A3F and selected with 1 μg/ml puromycin. 293T cells stably expressing HA-tagged A3G or A3F were generated by transduction with MLV based vectors expressing the proteins of interest and selection with 1 μg/ml puromycin. Expression levels of the A3 proteins were assessed by immunoblot using rabbit polyclonal sera specific for A3G [66] or A3F [67] for primary detection.

### Individual-nucleotide resolution UV cross-linking and immunoprecipitation

iCLIP has been described in detail previously [39]. Briefly, CEM-SS T cells stably expressing the proteins of interest were infected with *vif*-deficient HIV_IIIB_. 48 h later, a sample was collected to assess infection by intracellular p24^Gag^ staining and flow cytometry, confirming that at least 80% of cells were productively infected. The supernatant was collected, filtered through a 0.45 μm pore filter, and viruses isolated through a 20% sucrose cushion (wt/vol) at 21000 × g for 2 h at 4°C and resuspended in PBS. Cells were collected, washed 6 times with PBS and resuspended in PBS. Cells and viruses were then radiated with 400 mJ/cm^2^ using a Stratlinker 2400. Cells were pelleted by centrifugation and the supernatant discarded. Cells and viruses were then resuspended in 1 ml of lysis buffer (50 mM Tris-HCL, pH 7.4; 100 mM NaCl; 1% NP-40; 0.1% SDS; 0.5% sodium deoxycholate and protease inhibitor) and sonicated. 0.16 μg of RNase A were added to High RNase samples and 0.04 ng to the other samples. Tubes were incubated at 37°C for 3 min and added to protein G dynabeads previously incubated with anti-T7 antibody (Novagen) or anti-GFP antibody (Roche). The RNAs were dephosphorylated using Shrimp alkaline phosphatase (Promega) and a pre-adenylated linker was ligated to the 3’-end of RNAs on beads. RNAs were radiolabeled with P^32^-ϒ-ATP and separated using SDS-polyacrylamide gel electrophoresis, electrophoretically transferred to nitrocellulose and visualised on film. Pieces of the membrane containing the RNAs of interest were excised and resuspended in PK buffer (100 mM Tris-HCl pH 7.5, 50 mM NaCl, 10 mM EDTA) containing 2 mg/ml proteinase K. RNAs were isolated with phenol/chloroform (Ambion) and precipitated with 2.5 volumes of 100% ethanol, 0.1 volumes of sodium acetate (3 M, pH 5.5) and 0.5 μl of glycoblue. RNAs were then pelleted and reverse transcribed using barcoded primers. cDNAs were run on a TBE-urea polyacrylamide gel and products ranging from 70–85, 85–110 and >110 base pairs were excised. Nucleic acids were extracted and circularised. A primer that anneals to the linker previously ligated to the RNAs was used to create a double stranded region and this DNA was digested with BamHI. cDNA was then amplified by PCR and sequenced on one lane of an Illumina GA2 flow cell with 50 nucleotides run length. Data is available at ArrayExpress with the accession number E-MTAB-2700.

Before mapping reads, adapter sequences were removed, and the barcodes for each sample within each library were used to identify which sequence was immunoprecipitated from each protein. Mapping of the reads was performed against the human genome (version Hg19/ GRCh37) and HIV-1_IIIB_ (GenBank ID EU541617) genome using bowtie [41]. Reads that aligned to a single position on the human genome or HIV-1 genome with at most two mismatches were considered for analysis. Genomic annotations were then assigned based on gene annotations provided by Ensembl (v59). Reads were also aligned to human repeat sequences using repeat masker [42] and a database of consensus sequences provided by the software. A maximum of 3 mismatches were allowed.

### Viral particle production, packaging assays and RNA and protein quantifications

HIV-1 virions were produced by transfecting 293T cells with *vif*-deficient pNL4-3 or pIIIB using polyethyleneimine (PEI). Virus were then harvested 48 h later and filtered through a 0.45 μm pore filter. Lentiviral vectors were produced in 293T cells by transfecting the p8.91 packaging plasmid, a lentiviral vector and the VSVg envelope plasmid pMDG2.1 [43] at a ratio of 2:2:1 using PEI. Vectors were harvested 48 h after transfection and filtered. Viral particles were quantified by p24^Gag^ enzyme linked immunosorbent assay (Perkin Elmer). VLPs were produced by transfecting the packaging plasmid of interest and the Rev expression vector, at a ratio of 2:1. Wherever stated, HA- or T7-tagged GFP, A3G or A3F vectors were co-transfected at a ratio of 1:5 to the packaging plasmid. The supernatant was collected 48 h later, filtered through a 0.45μm pore filter and isolated through a 20% sucrose cushion (wt/vol) at 21000 × g for 2 h at 4°C. Cells and viral pellets were lysed in radioimmunoprecipitation assay (RIPA) buffer (50 mM Tris-HCl pH 7.4, 100 mM NaCl, 1 mM MgCl_2_, 1% NP-40, 0.1% SDS, 0.5% sodium deoxycholate). T7-tagged A3G was purified as described before [68]. Expression, VLP production and packaging of APOBEC3 proteins were assessed by standard immunoblot using anti-HA (mouse monoclonal; 12CA5) or anti-T7 (Novagen) and anti-p24^Gag^ (mouse monoclonal; p24-2 [69]) antibodies and detected and quantified by Li-cor Odyssey infrared imaging using IRDye800CW or IRDye680LT-labeled secondary antibodies. VLPs produced for RNA and protein quantification were filtered, layered over a continuous sucrose gradient (60–20%) and centrifugated at 150,000 × g for 1h15min at 4°C. 1mL fractions were collected and centrifugated at 21000 × g for 2 h at 4°C. The supernatant was removed and the pellet resuspended in RIPA buffer. The fractions containing p24^Gag^ were identified by immunoblot. The fraction with highest level of p24^Gag^ was then used to quantify the packaged A3G/F by immunoblot using a standard curve of purified T7-A3G. The remainder of the fraction was used to extract RNA using a microRNA extraction kit (Promega). Total RNA was then quantified using the Qubit RNA HS assay kit (Life Technologies), following the manufacturer’s instructions.

### Reverse transcription and quantitative PCR:

RNA was extracted from viral particles using Tri Reagent LS (Sigma) according to the manufacturer’s instructions. 0.5 μg of total RNA was used to synthesise cDNA with the high-capacity cDNA reverse transcription kit (Applied Biosystems) and random primers using the manufacturer’s protocol. qPCR was then performed using the primers 5′-TAACTAGGGAACCCACTGC-3′, 5′-GCTAGAGATTTTCCACACTG-3′ and the probe 5′-6-carboxyfluorescein [FAM]-ACACAACAGACGGGCACACACTA-6-carboxytetramethylrhodamine [TAMRA]-3′ to detect HIV-1 gRNA; SYBR Green (Applied Biosystems) was used to detect 7SL with the primers 5′-GGGCTGTAGTGCGCTATGC-3′ and 5′-CCCGGGAGGTCACCATATT-3′, and to quantify hY3 RNA with the primers 5′-GGCTGGTCCGAGTGCAGTG-3′ and 5′-AAAGGCTAGTCAAGTGAAGCAGTGG-3′.

## Supporting Information

S1 Fig Stable expression of tagged A3G and A3F in CEM-SS cells. Whole cell lysates of CEM-SS cells stably expressing T7-A3G, T7-A3F, GFP-A3G or GFP-A3F were compared to unmodified CEM cells by immunoblot analysis.
(TIF)

S2 Fig Intracellular p24^Gag^ staining of stable CEM-SS cells used for iCLIP. CEM-SS cells stably expressing proteins of interest were infected with *vif*-deficient HIV-1_IIIB_. Cells were harvested 48 h later for iCLIP analysis and a sample was used to analyse the infection. Intracellular p24^Gag^ expression was then assessed by flow cytometry, using uninfected cells as the negative control. Representative data are shown, with the percentages of positive cells highlighted within the boxed areas.
(TIF)

S3 Fig Restriction of HIV-1 by tagged A3G and A3F in stably expressing CEM-SS cells. CEM-SS cells stably expressing T7-GFP, T7-A3G, T7-A3F, GFP, GFP-A3G or GFP-A3F were infected with *vif*-deficient NL4-3. Cells were thoroughly washed at 24 h, and viruses harvested 48 h after infection. p24^Gag^ was measured by ELISA and virus stocks corresponding to 25 ng p24^Gag^ were used to infect TZM-BL reporter cells. Cell lysates were prepared 24 h later and assayed for luciferase activity. The graph shows the average of 3 independent experiments with standard deviations.
(TIF)

S4 Fig Schematic representation of Gag and Gag-Pol constructs. In the Gag ΔNC construct, NC was removed and replaced by the leucine zipper domain Zwt. The construct Gag ΔΨ lacks the packaging signal (specifically, SL2 and 3). RNA binding domains (RBD) derived from various proteins (SRP19, Ro, hnRNP C, hnRNP K, SRSF2 or Staufen-1) were fused to Gag ΔNC and are represented by Gag-RBD.
(TIF)

S5 Fig A3G and A3F mediated restriction of HIV-1 is not affected by reduced 7SL RNA packaging. 293T cells were transfected with expression vectors for HA-tagged GFP, A3G or A3F, and with SRP19 or an empty plasmid. All cultures were also co-transfected with the *vif*-deficient NL4-3 provirus. Ratios of the plasmids used for the transfection were 1:1:1. Viruses were harvested 48 h after transfection. p24^Gag^ was measured by ELISA and stocks corresponding to 25 ng p24^Gag^ were used to challenge TZM-BL cells. Cell lysates were harvested 24 h later and assayed for luciferase activity. The graph shows the average of 3 independent experiments with standard deviations.
(TIF)

S6 Fig Isolation of VLPs through sucrose gradients. 293T cells were co-transfected with plasmids coding for T7-A3G or T7-A3F, Gag and Rev. An irrelevant plasmid was transfected into cells serving as the negative control. VLPs were then recovered from the supernatant by centrifugation through a continuous sucrose gradient and fractionation. The figure shows representative immunoblots detecting Gag and packaged A3G/F.
(TIF)

S1 Table Percentage of reads aligning to specific regions of the human genome. (XLSX)

S2 Table Percentage of reads that aligned to non-coding RNAs. (XLSX)
