# Supplementary figures and images for: Promiscuous RNA Binding Ensures Effective Encapsidation of APOBEC3 Proteins by HIV-1
Source: PLoS Pathog. 2015 Jan 15;11(1):e1004609. doi: 10.1371/journal.ppat.1004609 (PMC4295846; doi:10.1371/journal.ppat.1004609)

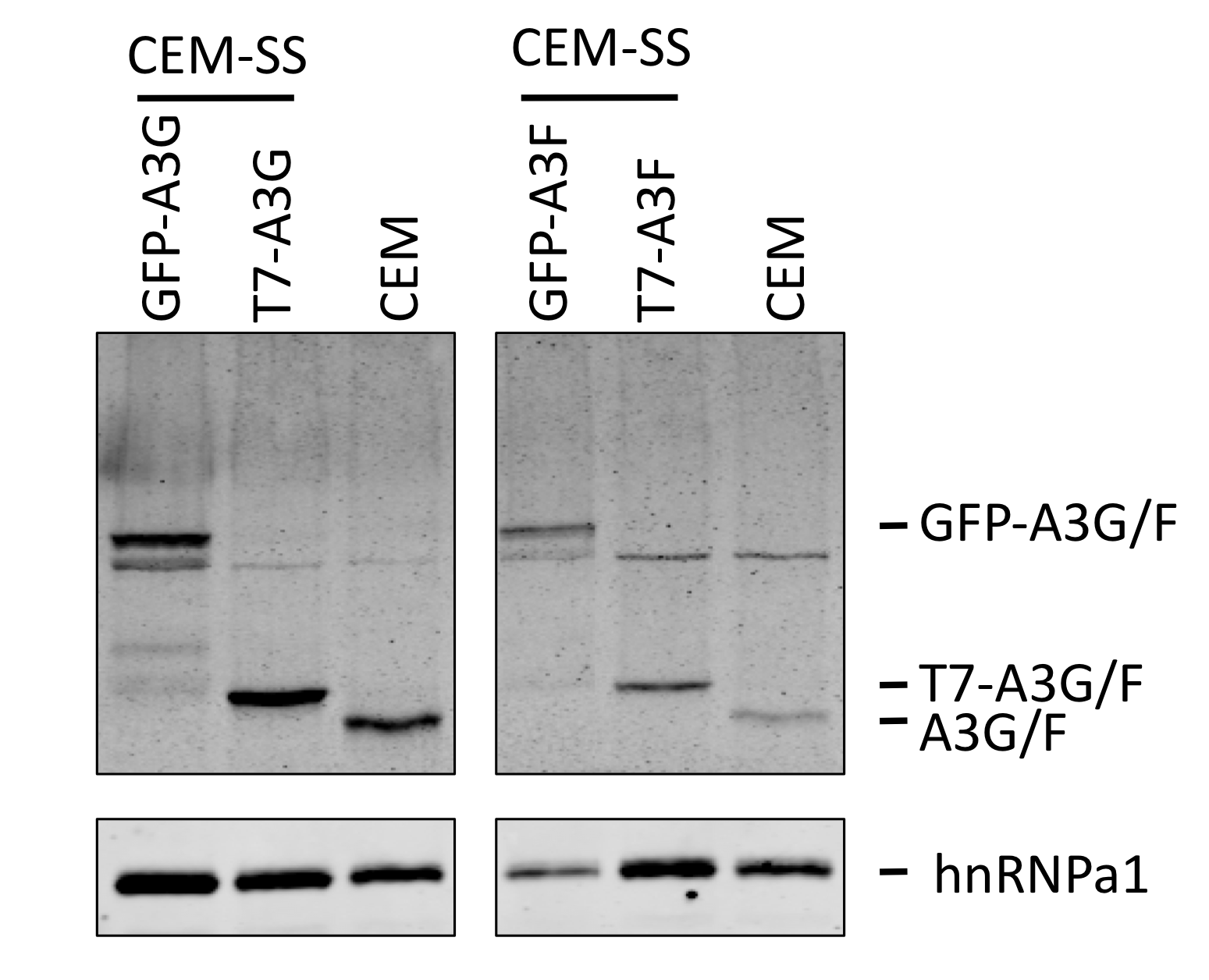

Supplement: S1 Fig — Whole cell lysates of CEM-SS cells stably expressing T7-A3G, T7-A3F, GFP-A3G or GFP-A3F were compared to unmodified CEM cells by immunoblot analysis. (TIF) [file ppat.1004609.s001.tif]

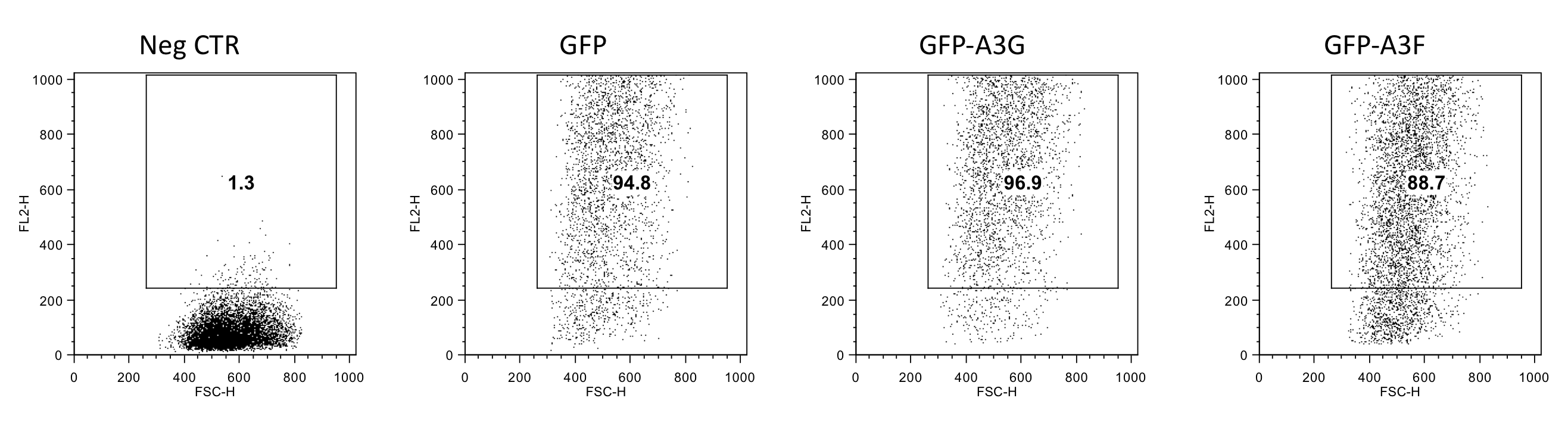

Supplement: S2 Fig — CEM-SS cells stably expressing proteins of interest were infected with vif-deficient HIV-1IIIB. Cells were harvested 48 h later for iCLIP analysis and a sample was used to analyse the infection. Intracellular p24Gag expression was then assessed by flow cytometry, using uninfected cells as the negative control. Representative data are shown, with the percentages of positive cells highlighted within the boxed areas. (TIF) [file ppat.1004609.s002.tif]

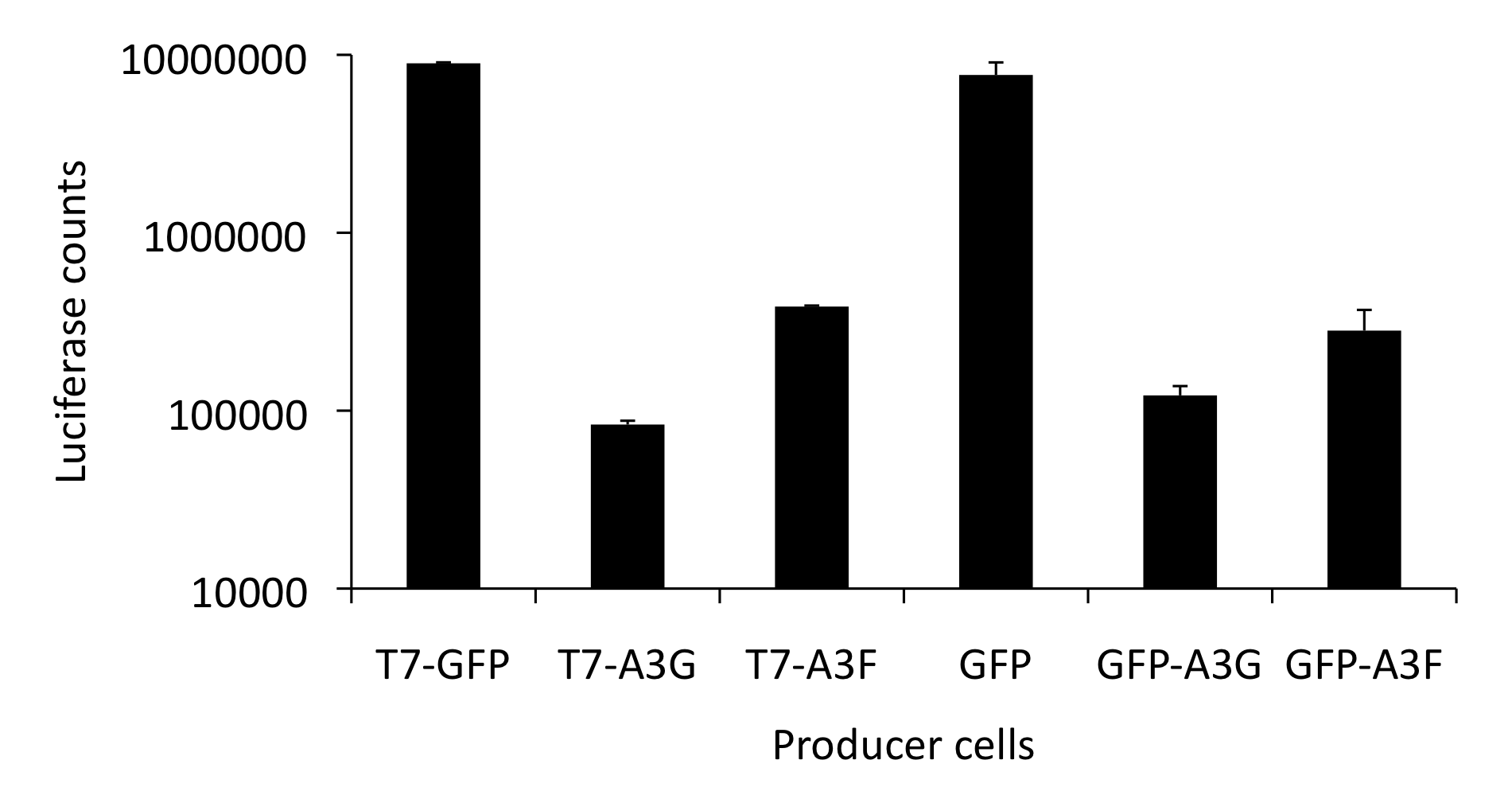

Supplement: S3 Fig — CEM-SS cells stably expressing T7-GFP, T7-A3G, T7-A3F, GFP, GFP-A3G or GFP-A3F were infected with vif-deficient NL4-3. Cells were thoroughly washed at 24 h, and viruses harvested 48 h after infection. p24Gag was measured by ELISA and virus stocks corresponding to 25 ng p24Gag were used to infect TZM-BL reporter cells. Cell lysates were prepared 24 h later and assayed for luciferase activity. The graph shows the average of 3 independent experiments with standard deviations. (TIF) [file ppat.1004609.s003.tif]

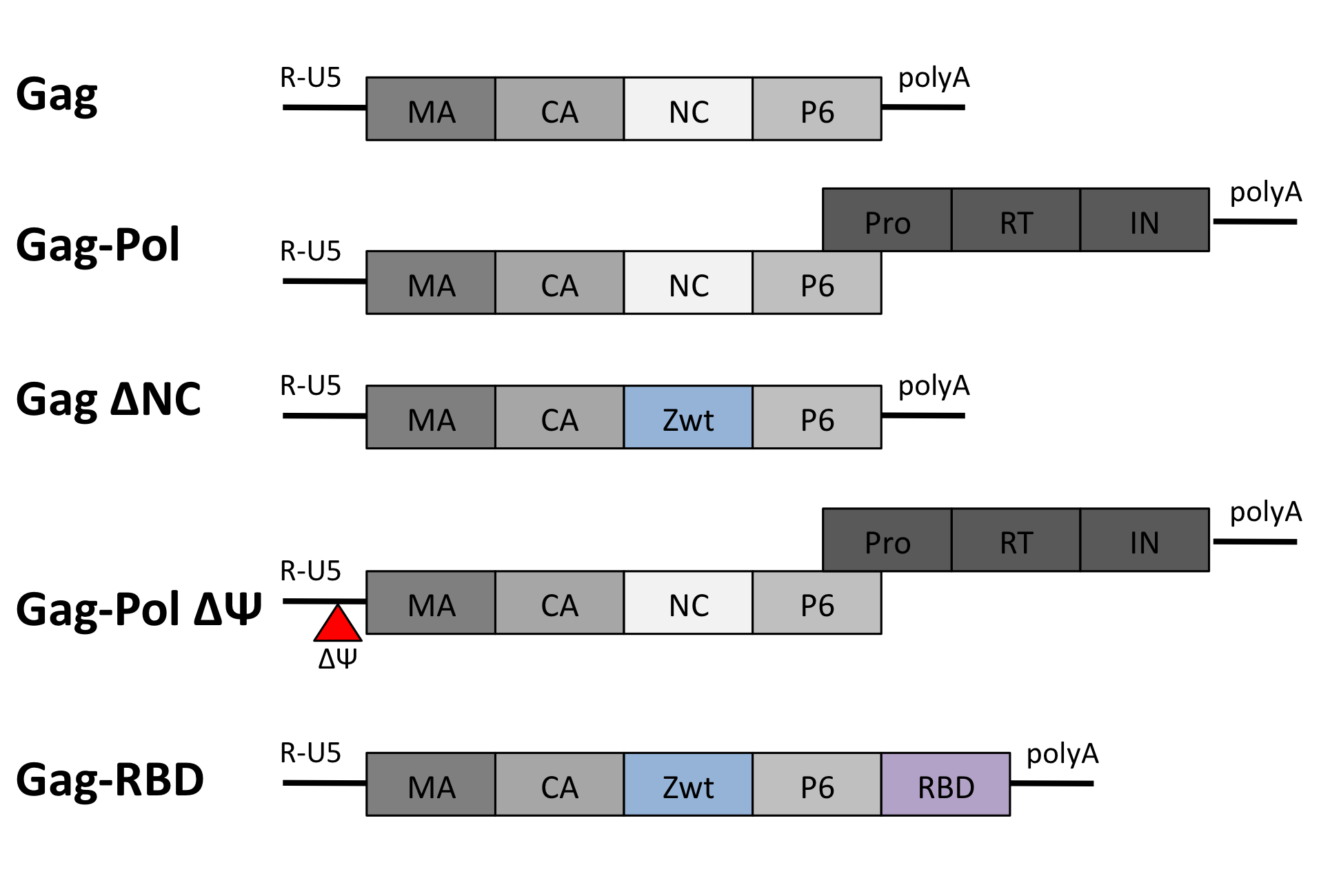

Supplement: S4 Fig — In the Gag ΔNC construct, NC was removed and replaced by the leucine zipper domain Zwt. The construct Gag ΔΨ lacks the packaging signal (specifically, SL2 and 3). RNA binding domains (RBD) derived from various proteins (SRP19, Ro, hnRNP C, hnRNP K, SRSF2 or Staufen-1) were fused to Gag ΔNC and are represented by Gag-RBD. (TIF) [file ppat.1004609.s004.tif]

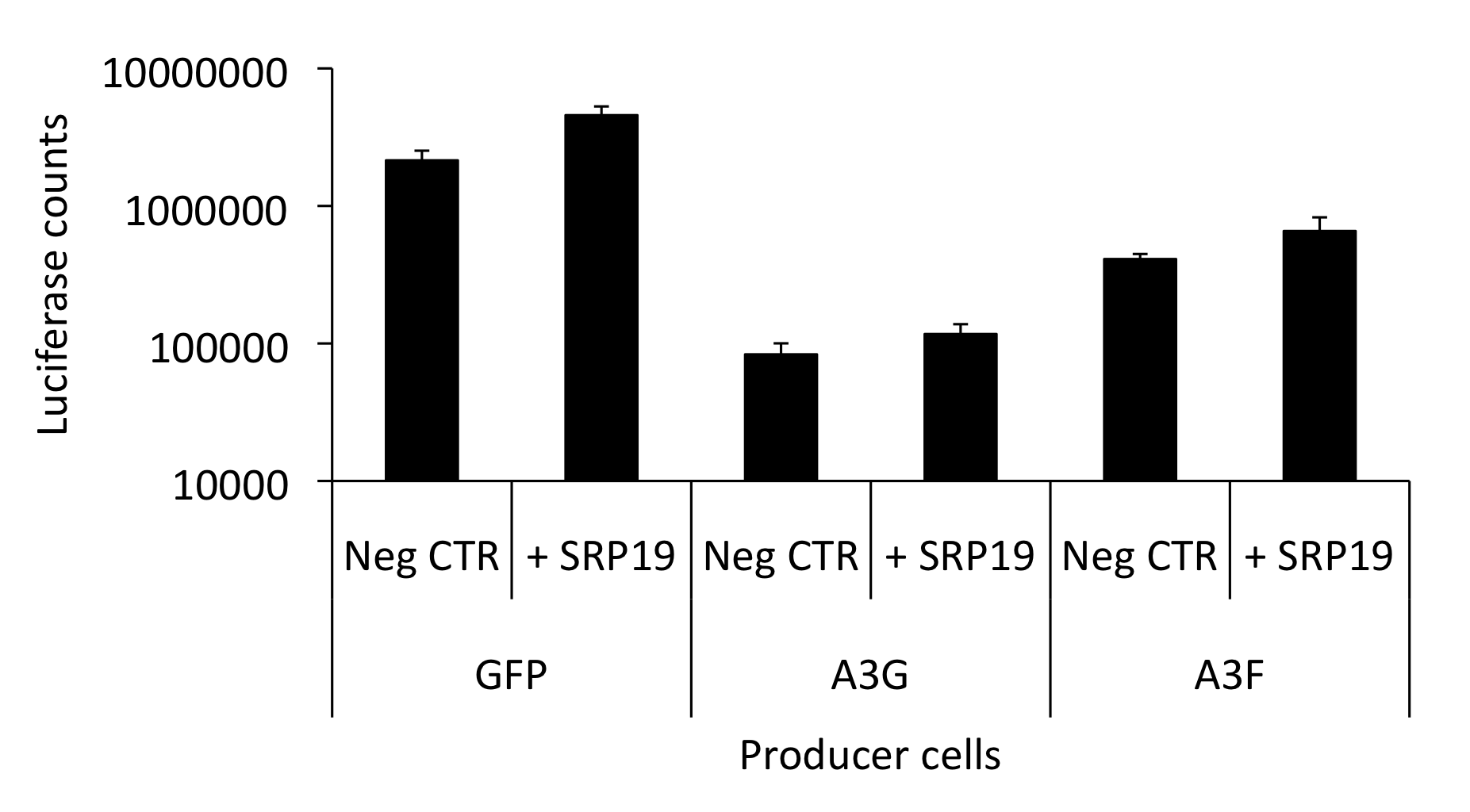

Supplement: S5 Fig — 293T cells were transfected with expression vectors for HA-tagged GFP, A3G or A3F, and with SRP19 or an empty plasmid. All cultures were also co-transfected with the vif-deficient NL4-3 provirus. Ratios of the plasmids used for the transfection were 1:1:1. Viruses were harvested 48 h after transfection. p24Gag was measured by ELISA and stocks corresponding to 25 ng p24Gag were used to challenge TZM-BL cells. Cell lysates were harvested 24 h later and assayed for luciferase activity. The graph shows the average of 3 independent experiments with standard deviations. (TIF) [file ppat.1004609.s005.tif]

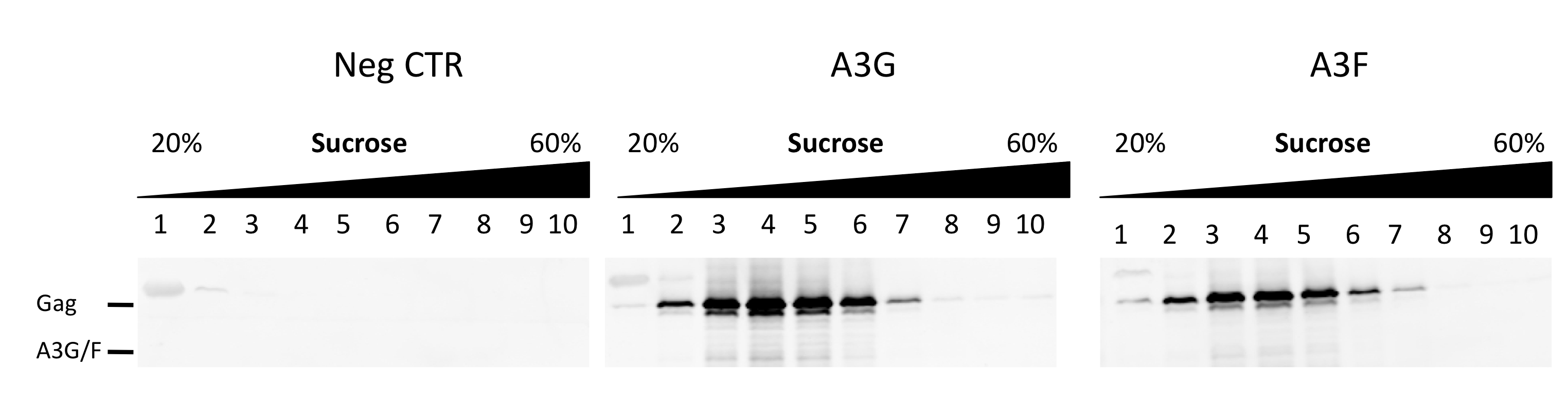

Supplement: S6 Fig — 293T cells were co-transfected with plasmids coding for T7-A3G or T7-A3F, Gag and Rev. An irrelevant plasmid was transfected into cells serving as the negative control. VLPs were then recovered from the supernatant by centrifugation through a continuous sucrose gradient and fractionation. The figure shows representative immunoblots detecting Gag and packaged A3G/F. (TIF) [file ppat.1004609.s006.tif]
